# Supplementary material for: Integration of FRET and sequencing to engineer kinase biosensors from mammalian cell libraries
Source: Nat Commun. 2021 Aug 19;12:5031. doi: 10.1038/s41467-021-25323-x (PMC8376904; doi:10.1038/s41467-021-25323-x)
Supplement: Supplementary file 1 — Supplementary Information [file 41467_2021_25323_MOESM1_ESM.pdf]

Supplementary Figures

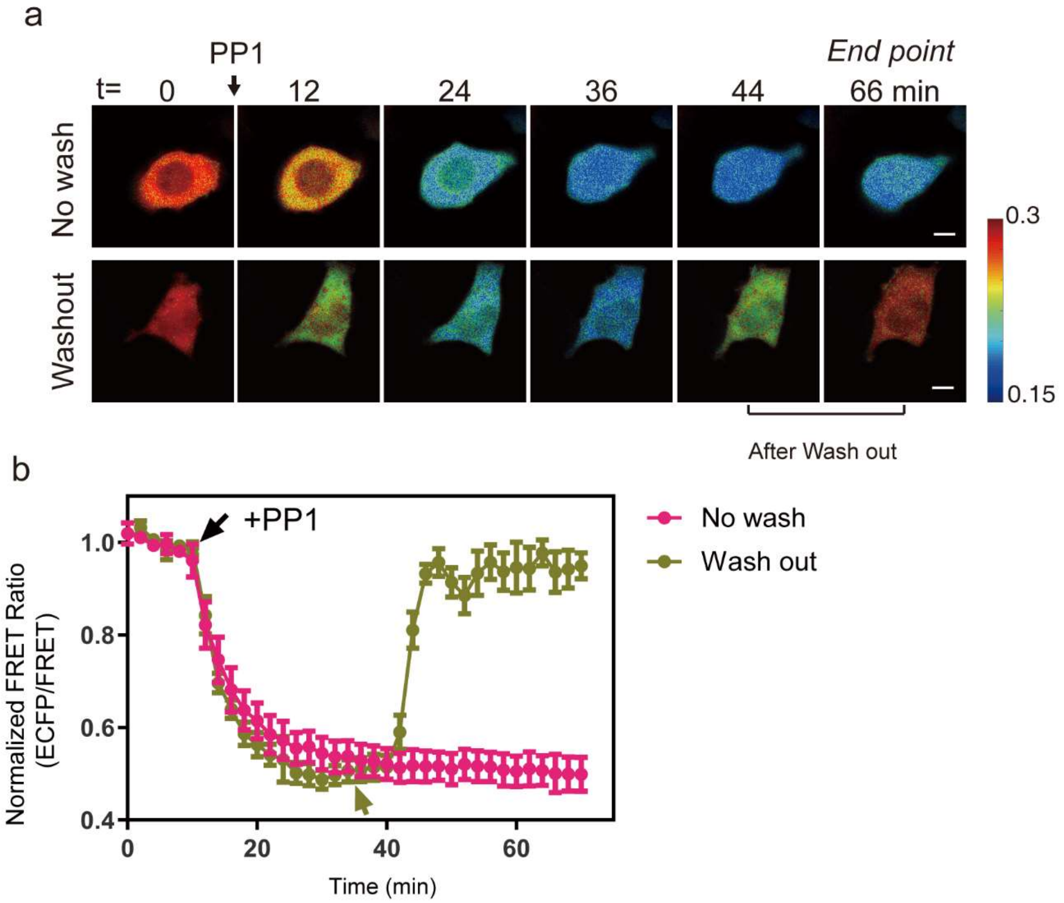

Supplementary Figure 1. The saFRET biosensor is reversible.

a-b, Representative images (a) and time courses (b) of the FRET (ECFP/FRET) ratio signals of the Fyn saFRET biosensors (Substrate: DYGYGVV) in different groups. PP1 was washed out during imaging in the wash-out group, while kept in the medium in the no-wash group (N=14 and 13, respectively). Error bars, mean ± SEM. Scale bars, 10 μm. Source data are provided as a Source Data file.

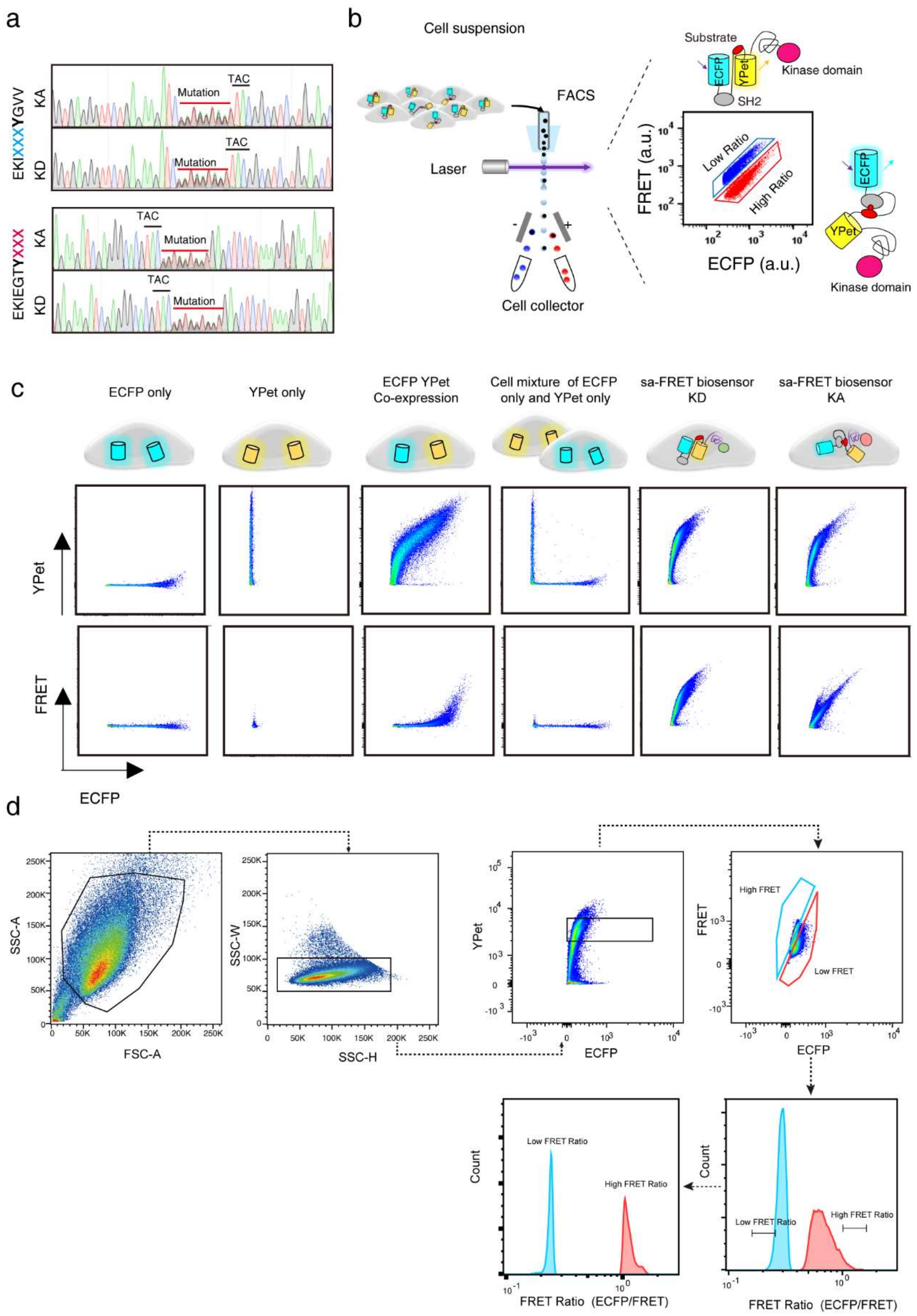

Supplementary Figure 2. Mammalian cell library screening by FACS

a, Sanger sequencing results showing random mutagenesis in the mutation region of the substrate peptide where EKIXXXYGVV represents library 1 (Lib1) with active (KA) or dead kinase (KD), and EKIEGTYXXX represents library 2 (Lib2) with active (KA) or dead kinase (KD). TAC encodes for

tyrosine.

b, Schematic of mammalian cell library screening by FACS. By using FACS, we can analyze the ECFP/FRET ratio of the FRET biosensor variants expressed in single cells.

c, Different control groups in FACS experiment. From left to right: only ECFP-expressing cells, only YPet-expressing cells, co-expression of ECFP- and YPet-expressing cells, mixture of only ECFP- or YPet-expressing cells, cells with KD FRET biosensor, cells with KA FRET biosensor. The top panel shows the relation between YPet intensity (y-axis) and ECFP intensity (x-axis); The bottom panel shows the relation between FRET intensity (y-axis) and ECFP intensity (x-axis).

d, Illustration of FACS experiment. After gate setting using the control biosensors in c, we analyzed and sorted the cells from different libraries. After single-cell gating, the cells with medium expression of FRET biosensor (as represented by YPet expression intensity) were gated and divided into High and Low ECFP/FRET ratio groups. Based on the ECFP/FRET ratio shown in the histogram plot, we can successfully separate the cells with different ratios (ECFP/FRET).

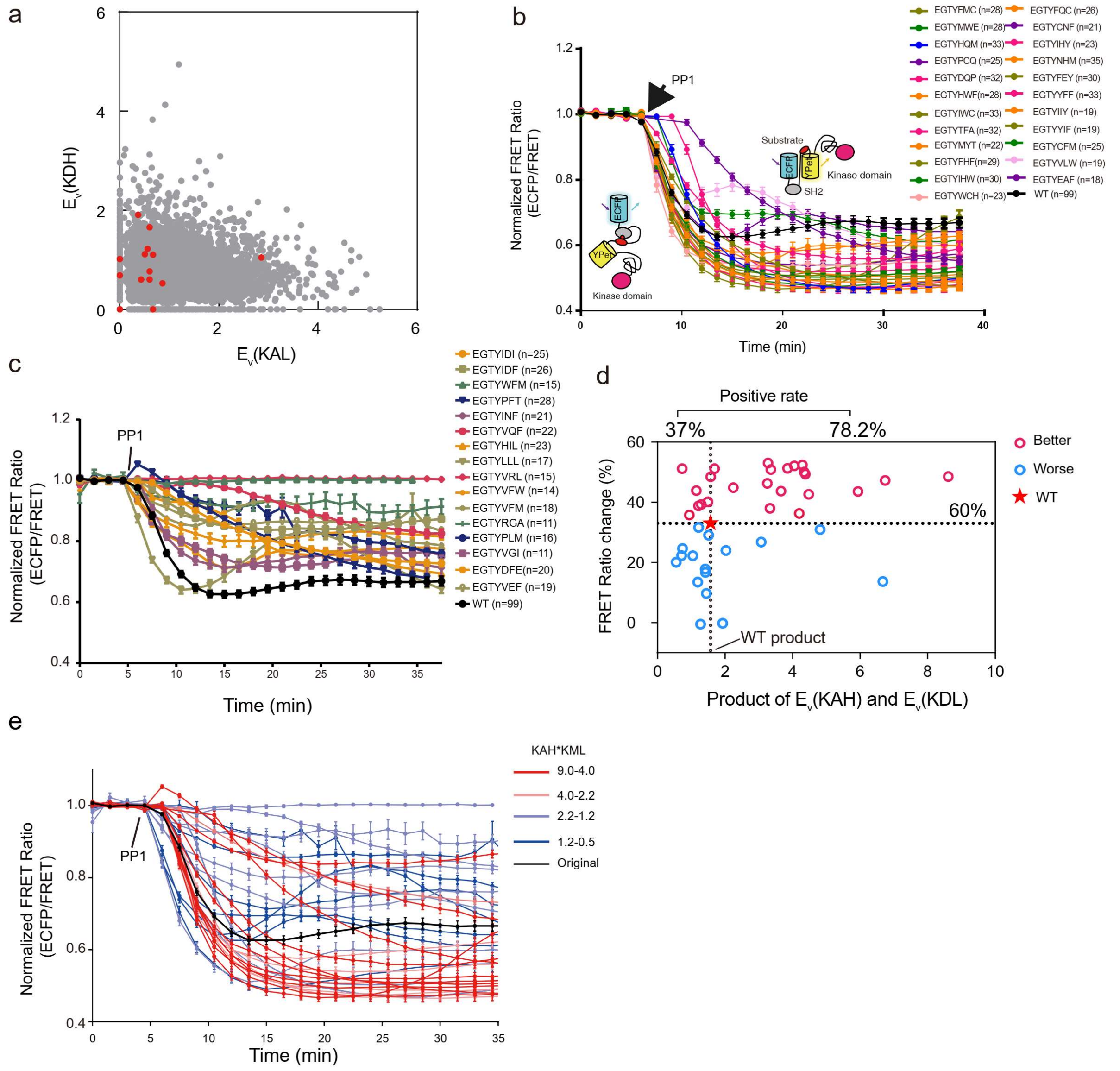

Supplementary Figure 3. The positive correlation of biosensors between the improved performance and the product of E_v(KAH) and E_v(KDL).

a, The desired biosensors identified were verified to be not enriched in either KAL or KDH group.

b, Quantification of the dynamic ECFP/FRET ratio of the better biosensor variants tested. The time course of ECFP/FRET ratio of the wild type biosensor before and after PP1 treatment was labeled as a black line (n is shown in the figure). Error bars, Mean ± SEM.

c, Quantification of the dynamic ECFP/FRET ratio of the worse biosensor variants tested. The time course of ECFP/FRET ratio of the wild-type biosensor before and after PP1 treatment was labeled as a black line (n is shown in the figure). Error bars, Mean ± SEM.

d. The relation between the experimentally verified dynamic range (%) of biosensor variants and their

product of E_v(KAH) and E_v(KDL). The dash lines represent the measured dynamic change (across y-axis) and the value of E_v(KAH)×E_v(KDL) (across x-axis) of wild-type biosensor. Red dots, blue dots, and the red star represent the better biosensor, worse biosensor, and WT biosensor, respectively. 37% and 78% represent the positive rates of identifying a better biosensor when the product values on X-axis are below or above that of the WT biosensor, respectively. 60% represents the overall positive rate without the WT biosensor as a reference.

e. The biosensors with different levels of E_v(KAH) ×E_v(KDL) were divided into four groups and their time courses accordingly colored with red, pink, light blue, and blue.

Source data are provided as a Source Data file.

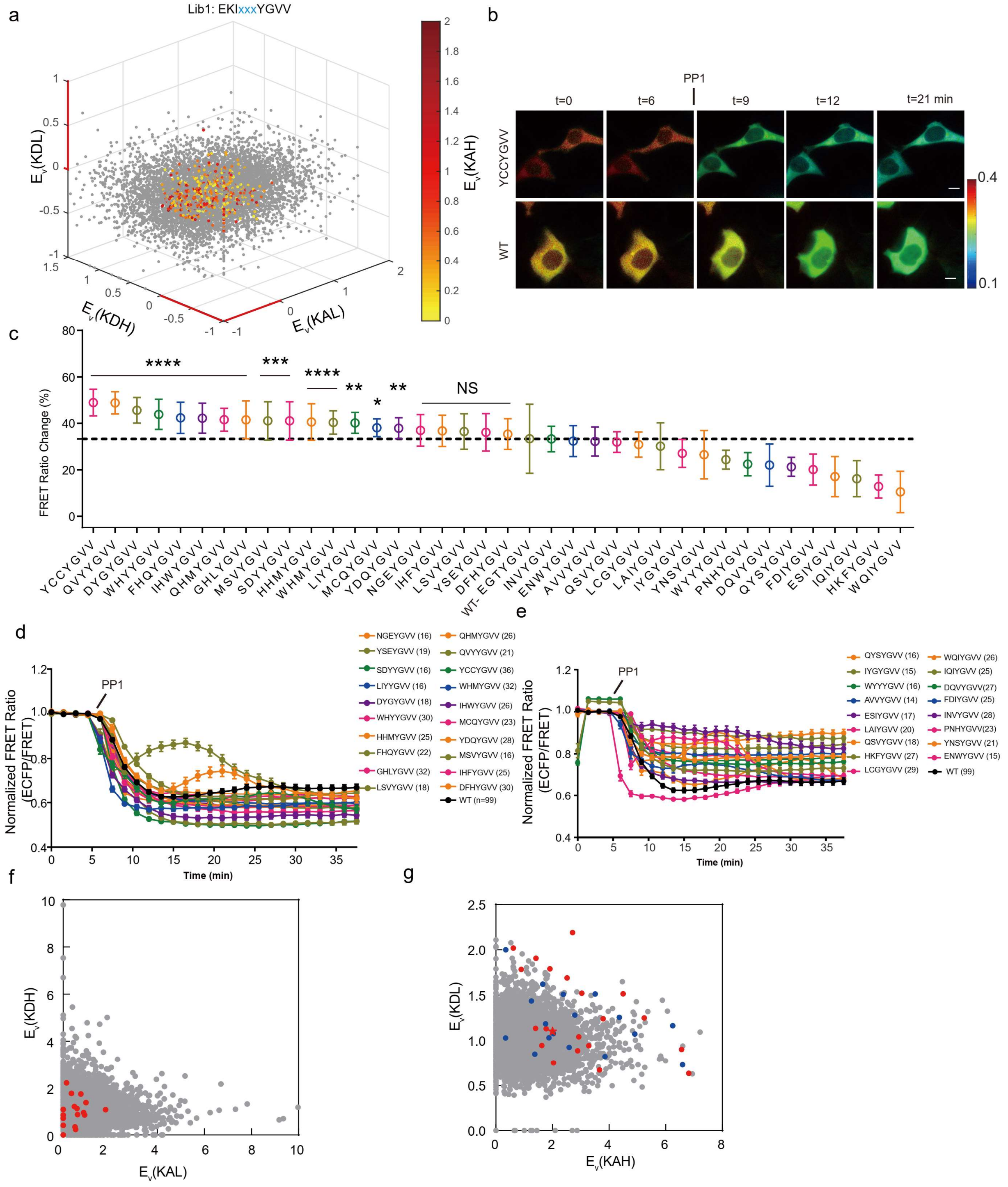

Supplementary Figure 4. The improvement of Fyn FRET biosensor via Lib1.

a, The 4D plot of the enrichment ratio (E_v) of substrates in different groups for Lib1 (XXXY), in which the amino acid residues before the consensus tyrosine were mutated. The enrichment ratio of the biosensors in the KAH group was color-coded. The substrates satisfying all four criteria were

highlighted in color.

b, Representative time-lapse images of the parental biosensor and one of the selected biosensors after PP1 treatment. Scale bars, 10 μm. The color bar represents the ECFP/FRET ratio, with hot and cold colors representing the high and low ratios, respectively.

c, Quantification of the FRET dynamic change (%) of selected biosensor variants upon PP1 treatment (From left to right, n= 36, 21, 18, 30, 22, 26, 26, 32, 16, 16, 25, 32, 16, 23, 28, 16, 25, 18, 19, 30, 99, 28, 15, 14, 18, 29, 20, 15, 21, 16, 23, 27, 16, 25, 17, 25, 27, and 26, respectively, One-way ANOVA, compared to the WT group, ****P<0.0001, ***P=0.0004 and 0.0004, **P=0.0016 and 0.0082, *p=0.0107, respectively, NS, not significant with P=0.0914, 0.0577, 0.1286, 0.1694, and 0.2288, respectively, only the variants with a mean value larger than the WT were subjected to statistical analysis). Error bars, mean ± SD.

d-e, Quantification of the normalized dynamic ECFP/FRET ratio of the better (d) and worse (e) biosensor variants that have been tested. FRET ratio change of the parental biosensor was marked in black line (n is shown in the figure). Error bars, Mean ± SEM.

f-g, Scatter plot of the enrichment ratio of biosensor variants. Red and blue dots represent biosensor variants with better and worse performance than the parental biosensor, respectively.

Source data are provided as a Source Data file.

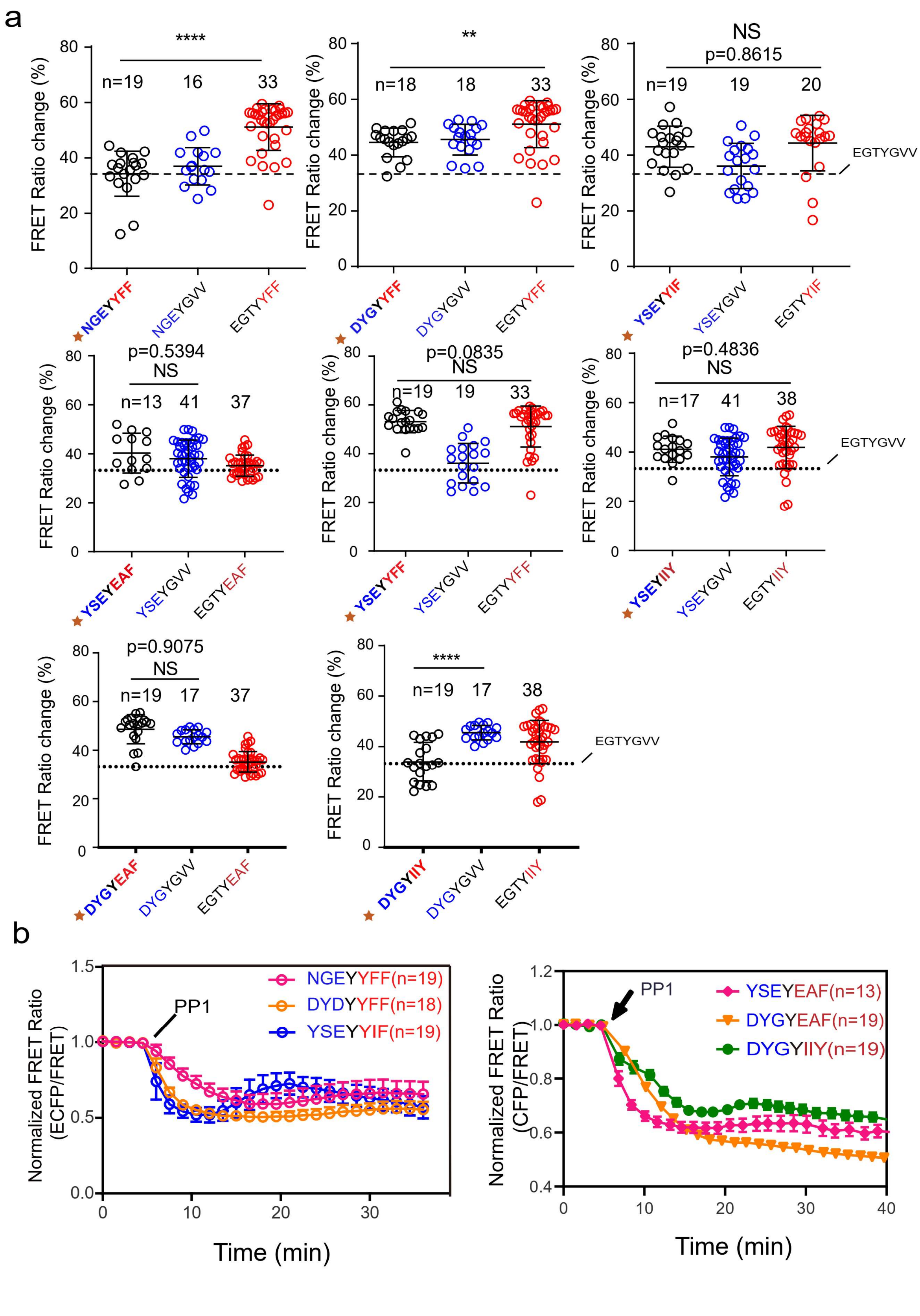

Supplementary Figure 5. The combination of two improved mutants from Lib 1 and Lib2.

a. Comparison of the biosensors with combined substrate sequences from both Lib 1 and Lib 2 vs their parental biosensors from either Lib 1 or Lib2. Star indicates the biosensors with combined substrate sequences (left columns). The middle columns are biosensors from Lib1 and the right columns from Lib2. The dashed lines indicate the mean FRET change of original WT (EGTYGVV) biosensor. (n is shown in the figure, One-way ANOVA, ****P < 0.0001, **P=0.0038, NS=Not significant and the corresponding p values are listed in the figures).

b. Time courses of the ECFP/FRET ratio signals of the combined biosensors after PP1 treatment. (n is shown in the figure, Error bars, Mean ± SD.

Source data are provided as a Source Data file.

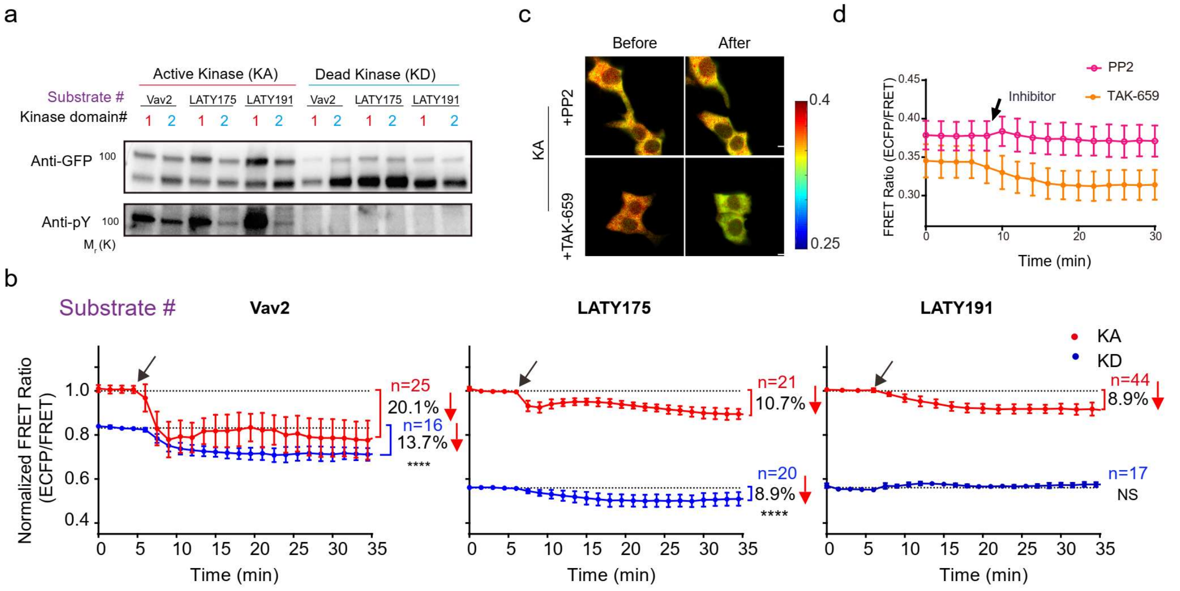

Supplementary Figure 6. Examining kinase domains and substrates for ZAP70 saFRET biosensor.

a, The effect of kinase domain on the biosensor phosphorylation. Kinase domain 1: ZAP70 327-619; and Kinase domain 2: ZAP70 327-601. Each biological replicate had similar results (n = 2).

b, Quantification of the dynamic ECFP/FRET ratio changes of ZAP70 saFRET biosensors with different substrates and kinase domain, upon the treatment by TAK-659 (25µM, black-arrow). Percentage indicates the reduction (red-arrow) of FRET ratio after TAK-659 treatment. Reduction of FRET ratio was observed in kinase dead biosensors with substrates from Vav2 and LATY175. (n is shown in the figure, Paired two-tailed t test, ****P<0.0001, NS, not significant with a P<0.05).

c-d, Representative images (c) and time courses (d) of the ECFP/FRET ratio signals of the ZAP70 saFRET biosensor with different inhibitors. TAK-659 (n=27). PP2, a Src family kinase inhibitor (n=13).

Error bars, mean ± SEM. Scale bars, 10 μm.

Source data are provided as a Source Data file.

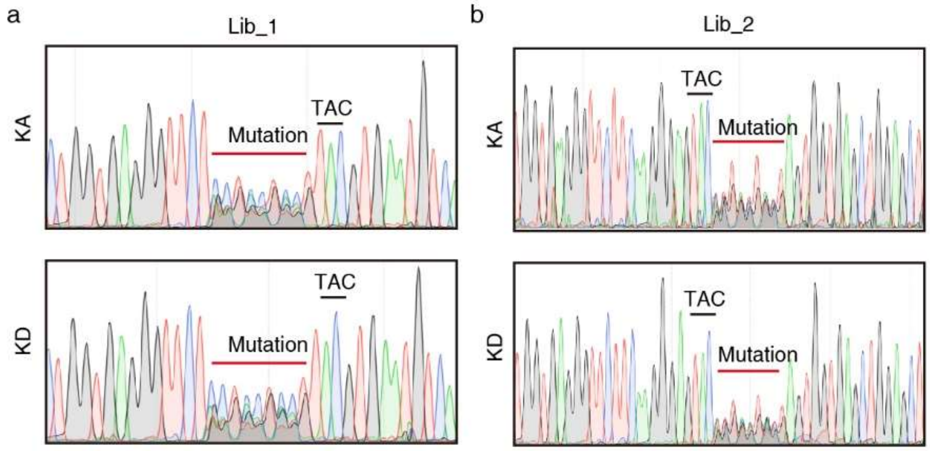

Supplementary Figure 7. Unbiased library generation for ZAP70 biosensor.

a, Sequencing results of library 1 (Lib1) with active (KA) or dead kinase (KD). TAC encodes for tyrosine.

b, Sequencing results of library 2 (Lib2) with active (KA) or dead kinase (KD).

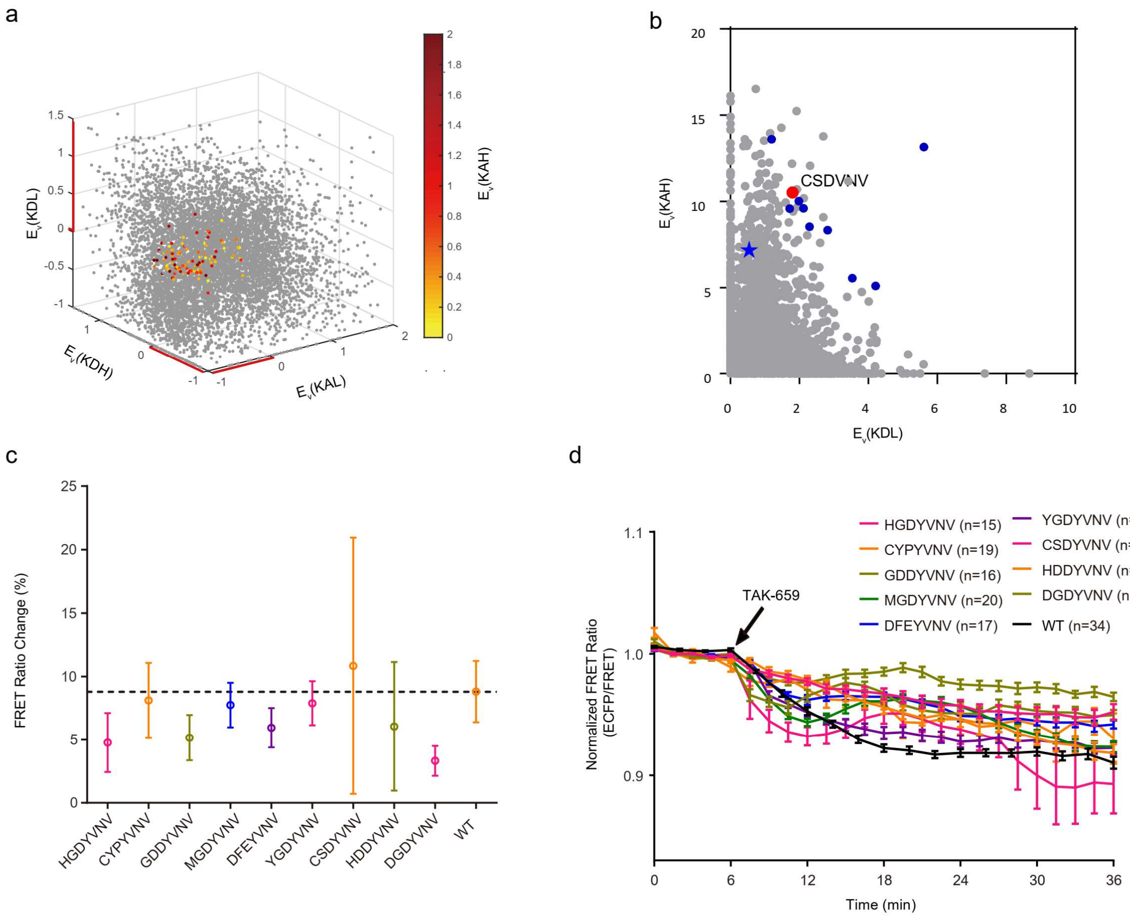

Supplementary Figure 8. The mutation of amino acid residues upstream to the consensus tyrosine in the substrate of the biosensors.

a, The 4D plot of the enrichment ratio (E_v) of substrates from different groups. The enrichment ratio in the KAH group was color-coded. The substrates satisfying all four criteria were highlighted with color.

b, Scatter plot of biosensors with different substrates. The biosensor variants with the top 10 products of E_v(KAH) and E_v(KDL) from Lib1 were labeled in Red (better biosensors than the parental biosensor) or Blue (worse biosensors than the parental biosensor).

c, Quantification of the dynamic change of biosensor variants upon PP1 treatment (From left to right, n= 15,19,16,20,17,20,19,15,15, and 34, respectively). Error bars, mean ± SD.

d, Quantification of the normalized dynamic ECFP/FRET ratio of the selected biosensor variants. FRET ratio change of the parental biosensor was marked in black line (n is shown in the figure). Error bars, Mean ± SEM.

Source data are provided as a Source Data file.

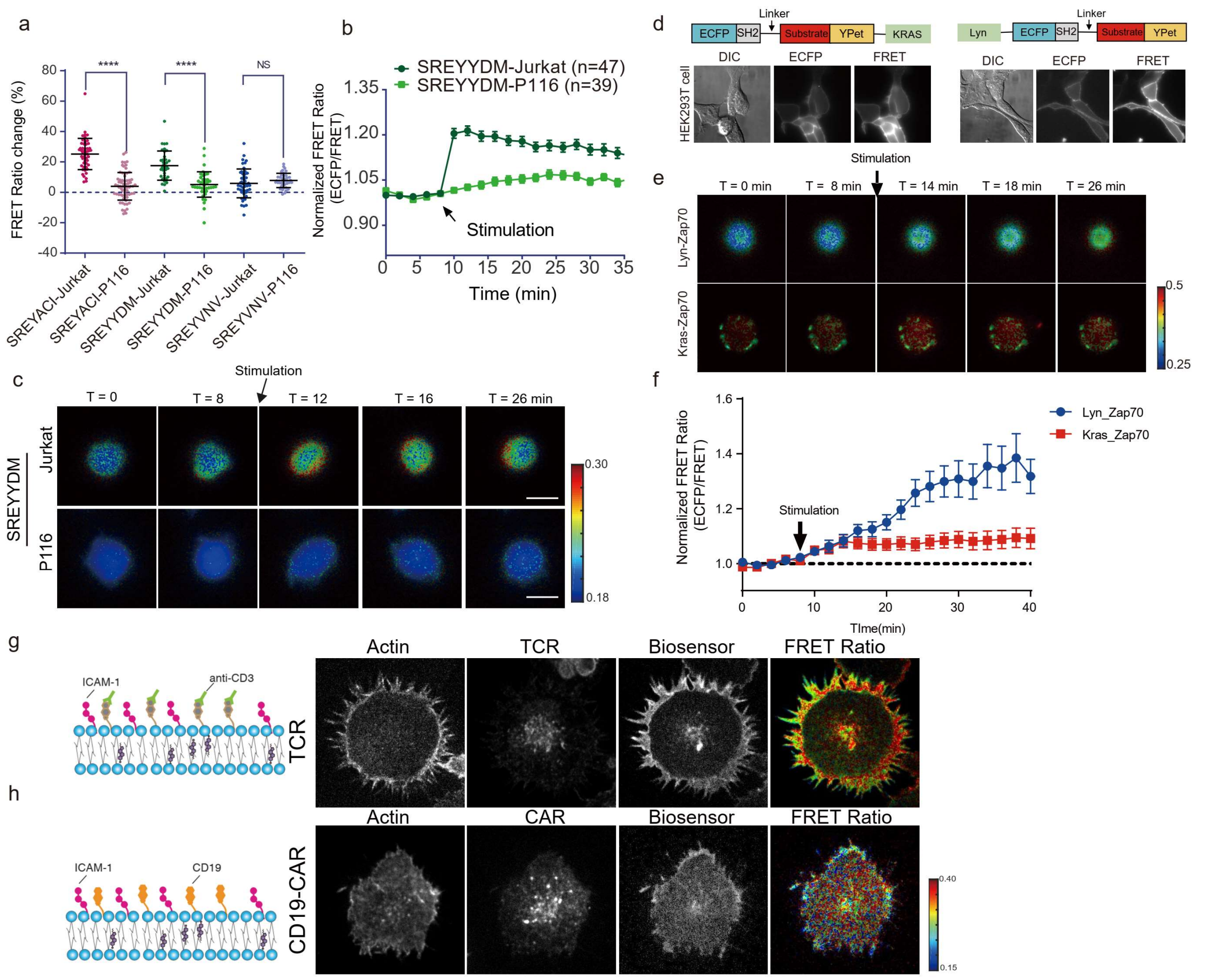

Supplementary Figure 9. Verification of the improved biosensors in primary human CD4+ T cells.

a, Dynamic ranges of the ZAP70 biosensors with different substrates. SREYVNV represents the parental biosensor (From left to right, n= 49,69,37,43,47, and 39, respectively). Jurkat, human T cell line. P116, ZAP70 $^{-/-}$ cell line derived from Jurkat cells. (Unpaired two-tailed Student's t-test, ****P<0.0001, NS, not significant, P=0.446).

b,c, Time courses (b) and time-lapse images (c) and of the SREYYDM biosensor before and after TCR activation induced by CD3/CD28 antibody stimulation (n is shown in the figure). Error bars, mean ± SD. Scale bars, 10 μm.

d, The design of the membrane-bound ZAP70 FRET biosensors and their membrane localization in HEK cells (representative of three experiments).

e, Representative time-lapse images of ZAP70 activity change in different membrane compartments after TCR activation in primary human T cells. Scale bars, 10 μm. The color bar indicates ECFP/FRET intensity ratio, with hot and cold colors representing the high and low ratios, respectively.

f, Time courses of normalized ECFP/FRET ratio of ZAP70 FRET biosensor in different membrane compartments (N=22 in each group). Error bars, mean ± SEM.

g. Schematics of solid supported lipid bilayer modified with anti-CD3 and ICAM-1 (the cartoon on the left). The images on the right show the spatial distributions of actin (labeled by LifeAct), TCR (by anti-CD3), ZAP70 localization (by biosensor intensity), and ZAP70 activity (by FRET ratio) in T cells, as indicated.

h. Schematics of solid supported lipid bilayer modified with CD19 and ICAM-1 (the cartoon on the left). The images on the right show the spatial distribution of actin (labeled by LifeAct), CAR (labeled by an anti-mouse IgG, F(ab')2), ZAP70 localization (by biosensor intensity), and ZAP70 activity (by FRET ratio) in T cells, as indicated.

Source data are provided as a Source Data file.

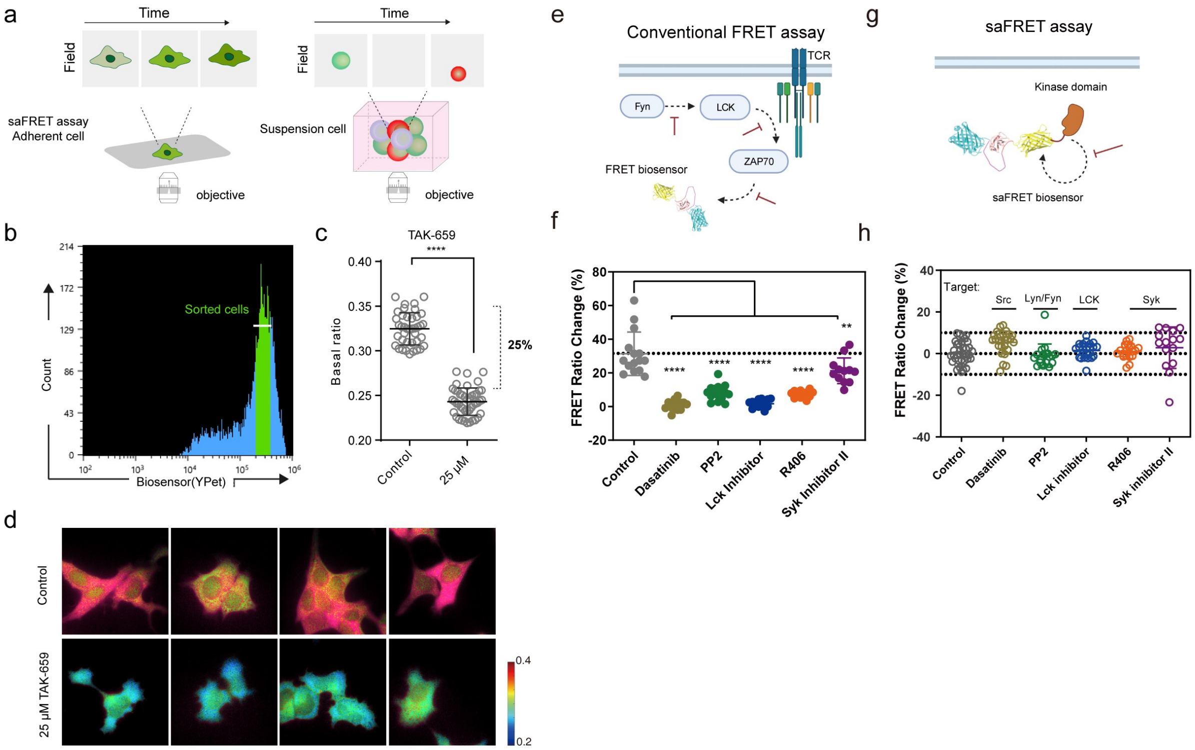

Supplementary Figure 10. Stable HEK 293T cell line with ZAP70 saFRET biosensor for HTDS assay targeting ZAP70 kinase.

a. Scheme illustrates the advantage of imaging adherent cells compared to suspension cells in general imaging platforms. Suspension cells, such as immune cells, float freely in media, and the focus or the observation field can easily become lost over time, especially at high magnification scale during imaging.

b, Cell sorting of the stable HEK293T cell line with a similar expression level of ZAP70 saFRET

biosensor. These sorted cells are used for HTDS assay.

c, The isolated stable HEK cell line expressing ZAP70 saFRET biosensor demonstrated ~25% change after a high-dose 25 μM TAK659 treatment (n=40 for each group, unpaired two-tailed Student's t test, ****P<0.0001).

d, Representative ECFP/FRET ratio images of ZAP70 saFRET biosensor after 25 μM TAK659 treatment in (c).

e, Schematics of conventional FRET assay in T cells. The FRET change could be affected by inhibitors targeting ZAP70 kinase and its upstream molecules.

f, The Src, Fyn, Lck, and Syk kinase inhibitors could reduce the FRET change of conventional biosensors after CD3/CD28 antibody stimulation significantly. The inhibitor groups are pretreated with corresponding inhibitors as indicated for 30 min before antibody stimulation. Control indicates the DMSO pre-treated group. (From left to right, n= 15,14,15,13,12, and 12, respectively, One-way ANOVA, ****P<0.0001 and **P=0.0013).

g, Schematics of saFRET assay in HEK293 cells. The FRET change is mediated by the kinase domain.

h, Inhibitors of Src, Fyn, Lck, and Syk kinases could not cause a significant FRET change of the ZAP70 saFRET biosensor. Control indicates the DMSO treated group. (From left to right, n= 36,27,18,23,19, and 15, respectively, and data were tested by One-way ANOVA).

Source data are provided as a Source Data file.

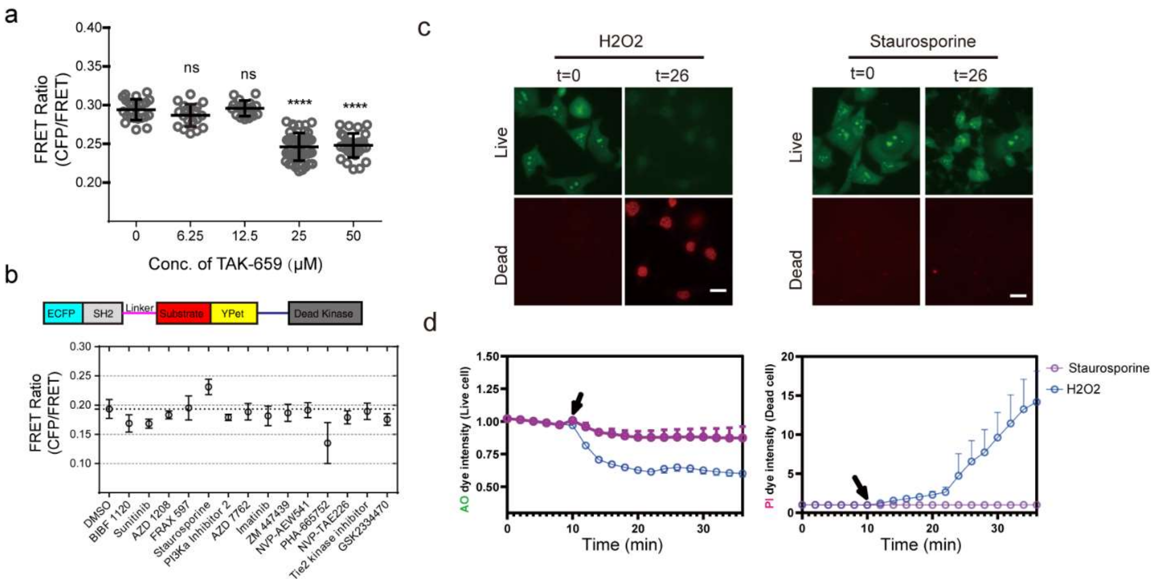

Supplementary Figure 11. HTDS using ZAP70 saFRET biosensors.

a, Concentration-dependent response of saFRET biosensor to TAK-659. The 10 μM TAK659 treatment

could not reduce the FRET ratio significantly. (From left to right, n= 41,28,19,41,30, respectively, unpaired two-tailed Student's t test, ****P<0.0001). Error bars, mean ± SD.

b, Top panel: The design of the ZAP70 biosensor with kinase-dead domain (saFRETkd). Bottom panel: The FRET ratio changes of a saFRET biosensor with kinase-dead domain in counter screening. Small molecules which have non-specific effects on FRET signals are eliminated in this step. (From left to right, n= 47 , 7, 26, 20, 25, 25, 14, 27, 15, 17, 17, 14, 17, 20, and 15, respectively).Error bars, mean ± SD.

c-d, Representative images (c) and time courses (d) of the green (live cell staining, AO dye) and red (dead cell staining, PI dye) channel signals of the cells under different treatments. H_2O_2 was used as a positive control to trigger cell death. No dead cell was observed during the time window of drug testing after the staurosporine treatment (10 µg/ml, same concentration to that in saFRET experiments). N=10 for each group. Error bars, mean ± SEM. Scale bars, 20 µm.

Source data are provided as a Source Data file.

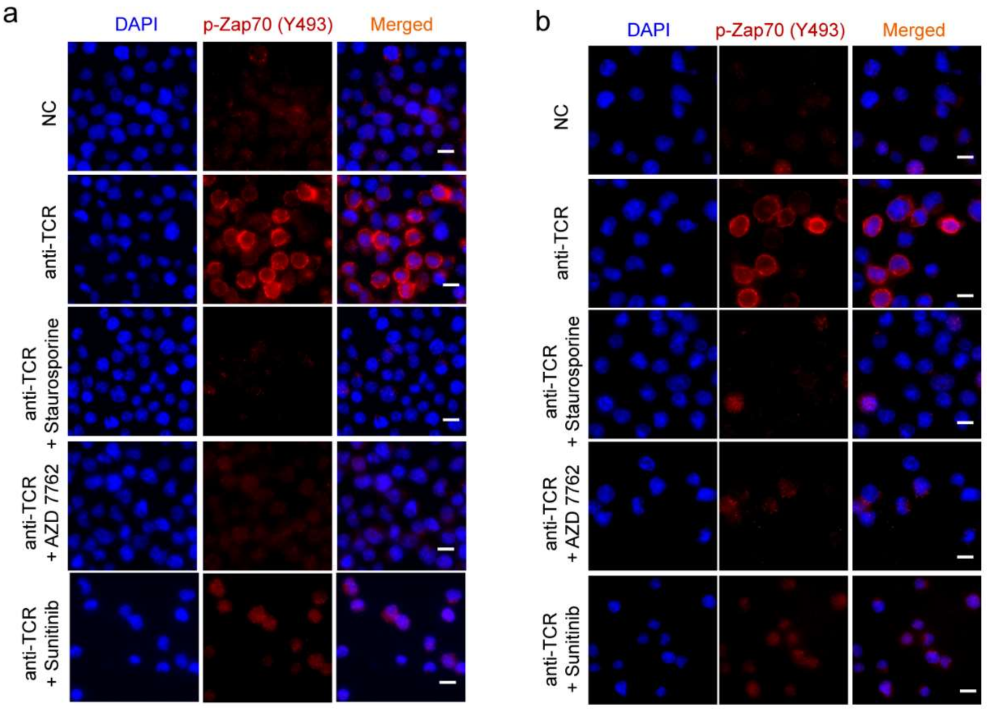

Supplementary Figure 12. Staurosporine and AZD7762 are potent inhibitors of ZAP70 signaling pathway.

a, Representative images of pZAP70 (Y493) in Jurkat T cells with different treatment. Scale bars, 10 µm. b, Representative images of pZAP70 (Y493) in P116-ZAP70-R360P cells with different

treatment. Scale bars, 10 μm. (a) and (b) were corresponding to the quantifications in Figure 7d and 7i, respectively.

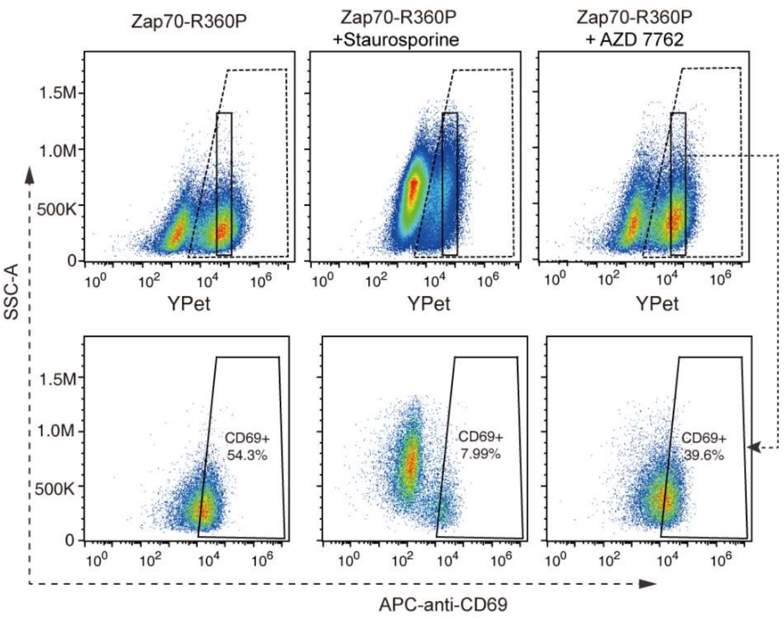

Supplementary Figure 13. Staurosporine and AZD 7762 inhibited the ZAP70 R360P mutation mediated T-cell activation

Flow-cytometric analysis of CD69 expression in P116–ZAP70-R360P cells with different inhibitor pre-treatment. ZAP70-WT or ZAP70-R360P expression levels were indicated by YPet intensity.
