# Supplementary material for: Integration of FRET and sequencing to engineer kinase biosensors from mammalian cell libraries
Source: Nat Commun. 2021 Aug 19;12:5031. doi: 10.1038/s41467-021-25323-x (PMC8376904; doi:10.1038/s41467-021-25323-x)
Supplement: Supplementary file 2 — Reporting Summary [file 41467_2021_25323_MOESM2_ESM.pdf]

nature research

Corresponding author(s): Yingxiao Wang

Last updated by author(s): 2021-07-14

Reporting Summary

Nature Research wishes to improve the reproducibility of the work that we publish. This form provides structure for consistency and transparency in reporting. For further information on Nature Research policies, see our Editorial Policies and the Editorial Policy Checklist.

Statistics

For all statistical analyses, confirm that the following items are present in the figure legend, table legend, main text, or Methods section.

n/a	Confirmed	
☐	☒	The exact sample size (*n*) for each experimental group/condition, given as a discrete number and unit of measurement
☐	☒	A statement on whether measurements were taken from distinct samples or whether the same sample was measured repeatedly
☐	☒	The statistical test(s) used AND whether they are one- or two-sided<br>*Only common tests should be described solely by name; describe more complex techniques in the Methods section.*
☒	☐	A description of all covariates tested
☒	☐	A description of any assumptions or corrections, such as tests of normality and adjustment for multiple comparisons
☐	☒	A full description of the statistical parameters including central tendency (e.g. means) or other basic estimates (e.g. regression coefficient) AND variation (e.g. standard deviation) or associated estimates of uncertainty (e.g. confidence intervals)
☐	☒	For null hypothesis testing, the test statistic (e.g. *F*, *t*, *r*) with confidence intervals, effect sizes, degrees of freedom and *P* value noted<br>*Give P values as exact values whenever suitable.*
☒	☐	For Bayesian analysis, information on the choice of priors and Markov chain Monte Carlo settings
☒	☐	For hierarchical and complex designs, identification of the appropriate level for tests and full reporting of outcomes
☒	☐	Estimates of effect sizes (e.g. Cohen's *d*, Pearson's *r*), indicating how they were calculated

Our web collection on statistics for biologists contains articles on many of the points above.

Software and code

Policy information about availability of computer code

Data collection	For collectiing imaging data, MetaFluor 7.8 or MetaMorph 7.8 software (Molecular Devices) were used. Other data collection methods were described in the Supplementary method section.
Data analysis	All statistical analyses were performed using GraphPad Prism version 9 or Matlab R2019b. FACS data was analyzed using FlowJo 10. Fluocell version V6.0.0 was used to analyze the FRET imaging data and the source code is available on a GitHub site: http://github.com/lu6007/. fluocell. Mutseq Version V1.10b was used to analyze the next-generation sequencing data of the library and the source code is available on a Github site: https://github.com/jason8301/mutseq.

For manuscripts utilizing custom algorithms or software that are central to the research but not yet described in published literature, software must be made available to editors and reviewers. We strongly encourage code deposition in a community repository (e.g. GitHub). See the Nature Research guidelines for submitting code & software for further information.

Data

Policy information about availability of data

All manuscripts must include a data availability statement. This statement should provide the following information, where applicable:

- Accession codes, unique identifiers, or web links for publicly available datasets
- A list of figures that have associated raw data
- A description of any restrictions on data availability

All data is available in the main text or the supplementary materials.

Field-specific reporting

Please select the one below that is the best fit for your research. If you are not sure, read the appropriate sections before making your selection.

☒ Life sciences ☐ Behavioural & social sciences ☐ Ecological, evolutionary & environmental sciences

For a reference copy of the document with all sections, see nature.com/documents/nr-reporting-summary-flat.pdf

Life sciences study design

All studies must disclose on these points even when the disclosure is negative.

Sample size	Sample size was determined based on similar studies (https://doi.org/10.1038/s41467-017-00569-6, https://doi.org/10.1038/s41556-018-0200-6) to obtain the convincing and compelling results. We have listed the exact sample size (n) for each experimental group in the manuscript.
Data exclusions	No data was excluded.
Replication	For Library screening of improved FRET biosensor, we have reproduced the experiment twice (i.e., Fyn and ZAP70 kinase) to ensure reliability and reproducibility. The improved biosensors were further tested successfully in at least three biological replicates. For all the other experiments, replication attempts (at least three biological replicates) were successful.
Randomization	The starting materials for each experiment were randomly assigned to different experiment treatments (e.g., biosensor transfection, stimulation or drug treatment)
Blinding	Investigators were not blinded to group allocation during data collection and analysis, because 1) the investigator determined the experiment conditions before each experiment, 2) all the samples were randomly assigned to different groups and treated in the same way to eliminate the bias during experiments, 3) the investigator did not know the results until only after they finish the experiment and objectively analyzed the data.

Reporting for specific materials, systems and methods

We require information from authors about some types of materials, experimental systems and methods used in many studies. Here, indicate whether each material, system or method listed is relevant to your study. If you are not sure if a list item applies to your research, read the appropriate section before selecting a response.

Materials & experimental systems

n/a	Involved in the study	
☐	☒	Antibodies
☐	☒	Eukaryotic cell lines
☒	☐	Palaeontology and archaeology
☒	☐	Animals and other organisms
☐	☒	Human research participants
☒	☐	Clinical data
☒	☐	Dual use research of concern

Methods

n/a	Involved in the study	
☒	☐	ChIP-seq
☐	☒	Flow cytometry
☒	☐	MRI-based neuroimaging

Antibodies

Antibodies used	Phospho-Zap70 (Tyr493)/Syk (Tyr526) #2704 from CST; phospho-LAT (Tyr191) #MA5-33177 from thermofisher; Alexa Fluor-594 donkey anti-Rabbit IgG#A21207 from Thermo Fisher Scientific; Anti-GFP antibody # ab290 from Abcam ; Anti-Phosphotyrosine antibody # 05321, clone 4G10 from Upstate;Mouse Anti-Human CD3, #555337, Clone HIT3a, BD biosciences;Mouse Anti-Human CD28,#556620,Clone CD28.2,BD biosciences; Anti-T-Cell Receptor Antibody, #05-919, clone C305,MilliporeSigma;<br>APC anti-human CD69 Antibody,#310909,clone FN50,Biolegend.
Validation	No customized antibodies were used. All antibodies used were validated for the specific application and species by the manufacturers. The validation data and references were provided in the manufacturer's website.<br><br>Phospho-Zap70 (Tyr493)/Syk (Tyr526) #2704<br>https://www.cellsignal.com/products/primary-antibodies/phospho-zap-70-tyr493-syk-tyr526-antibody/2704<br><br>phospho-LAT (Tyr191) #MA5-33177<br>https://www.thermofisher.com/antibody/product/Phospho-LAT-Tyr191-Antibody-Recombinant-Monoclonal/MA5-33177<br><br>Alexa Fluor-594 donkey anti-Rabbit IgG#A21207<br>https://www.thermofisher.com/antibody/product/Donkey-anti-Rabbit-IgG-H-L-Highly-Cross-Adsorbed-Secondary-Antibody-Polyclonal/A-21207

Anti-GFP antibody (ab290)
https://www.abcam.com/gfp-antibody-ab290.html

Anti-Phosphotyrosine antibody # 05321, clone 4G10 from Upstate)
https://www.sigmaaldrich.com/US/en/product/mm/05321?context=product

Mouse Anti-Human CD3,BD biosciences
https://www.bdbiosciences.com/en-us/products/reagents/flow-cytometry-reagents/research-reagents/single-color-antibodies-ruo/purified-mouse-anti-human-cd3.555337

Mouse Anti-Human CD28,BD biosciences
https://www.bdbiosciences.com/en-us/products/reagents/flow-cytometry-reagents/research-reagents/single-color-antibodies-ruo/purified-mouse-anti-human-cd28.556620

Anti-T-Cell Receptor Antibody clone C305,MilliporeSigma
https://www.sigmaaldrich.com/US/en/product/mm/05919

APC anti-human CD69 Antibody,BioLegend
https://www.biolegend.com/en-us/search-results/apc-anti-human-cd69-antibody-1674

Eukaryotic cell lines

Policy information about cell lines

Cell line source(s)	Lenti-X 293T (#632180, Clontech Laboratories), HEK293 cells (#CRL-1573,ATCC),Jurkat (Clone E6-1, #TIB-152,ATCC) ,P116 (CRL-2676,ATCC),Raji (#CCL-86, ATCC),NIH-3T3 (#CRL-1658, ATCC)
Authentication	Cell lines were verified by the manufacturer.ATCC using morphology, karyotyping, and STR profiling to confirm the identity of human cell lines. Lenti-X 293T cells was used to produce lentivirus, thus it is not directly involved in this study.<br>HEK293<br>https://www.atcc.org/products/crl-1573#detailed-product-information<br>Jurkat E6<br>https://www.atcc.org/products/tib-152<br>P116 cell<br>https://www.atcc.org/products/crl-2676#detailed-product-information<br>Raji<br>https://www.atcc.org/products/ccl-86<br>NIH-3T3<br>https://www.atcc.org/products/crl-1658
Mycoplasma contamination	All cell lines were tested negative for mycoplasma contamination.
Commonly misidentified lines (See ICLAC register)	No commonly misidentified lines were used in this study.

Human research participants

Policy information about studies involving human research participants

Population characteristics	Peripheral blood mononuclear cells (PBMCs) were isolated from blood samples of healthy donors from San Diego Blood Bank(SDBB) after donor de-identification.
Recruitment	No potential self-selection bias is known since PBMCs were isolated from donors of SDBB after donor de-identification.
Ethics oversight	This sample collection is exempted from Institutional Review Board (IRB); thus, this study was not considered to involve human research participants according to Human Research Protection Program of University of California, San Diego, CA, USA. (https://irb.ucsd.edu/Exemption_fact_sheet.pdf, #4)

Note that full information on the approval of the study protocol must also be provided in the manuscript.

Flow Cytometry

Plots

Confirm that:

☒ The axis labels state the marker and fluorochrome used (e.g. CD4-FITC).

☒ The axis scales are clearly visible. Include numbers along axes only for bottom left plot of group (a 'group' is an analysis of identical markers).

☒ All plots are contour plots with outliers or pseudocolor plots.

☒ A numerical value for number of cells or percentage (with statistics) is provided.

Methodology

Sample preparation	Cells from different groups, as described in Supplementary figure 1 and Supplementary method section, were trypsinized and washed with cell culture medium and PBS. Cells were then suspended in PBS + 5% BSA.
Instrument	BD FACS Aria II cell sorter (BD bioscience)
Software	FlowJo Software (FlowJo LLC)
Cell population abundance	At least 200,000 counts were sorted in the library screening experiment.
Gating strategy	Doublets, dead cells, and non-infected cells were excluded by gating on WT cells. To analysis the FRET signal, several control groups were used and the detailed gating methods were listed in supplementary Figure 1 and supplementary method section.

☒ Tick this box to confirm that a figure exemplifying the gating strategy is provided in the Supplementary Information.
