# Supplementary material for: Integration of FRET and sequencing to engineer kinase biosensors from mammalian cell libraries
Source: Nat Commun. 2021 Aug 19;12:5031. doi: 10.1038/s41467-021-25323-x (PMC8376904; doi:10.1038/s41467-021-25323-x)
Supplement: Supplementary file 18 — Description of Additional Supplementary Files [file 41467_2021_25323_MOESM18_ESM.pdf]

Title: Supplementary data 1:

Description: Frequency and the enrichment ratio of Fyn biosensor variants in library 2.

Title: Supplementary data 2:

Description: FRET ratio changes of selected biosensor variants in Fyn library 2.

Title: Supplementary data 3:

Description: Frequency and the enrichment ratio of Fyn biosensor variants in library 1.

Title: Supplementary data:

Description: FRET ratio changes of selected biosensor variants in Fyn library 1.

Title: Supplementary data 5:

Description: Frequency and the enrichment ratio of ZAP70 biosensor variants in library 2.

Title: Supplementary data 6:

Description: Frequency and the enrichment ratio of ZAP70 biosensor variants in library 1.

Title: Supplementary data 7:

Description: Primers used in this study.

Title: Supplementary Video 1:

Description: Time course of Fyn-saFRET biosensors with active kinase domain (KA) or dead kinase domain (KD) before and after PP1 treatment. Scale bars, 10 µm. The color bar indicates ECFP/FRET emission ratio, with hot and cold colors representing the high and low ratios, respectively.

Title: Supplementary Video 2:

Description: Time course of the parental and improved Fyn biosensors before and after PP1 treatment. Scale bars, 10 µm. The color bar indicates ECFP/FRET ratio, with hot and cold colors representing the high and low ratios, respectively.

Title: Supplementary Video 3:

Description: Time course of ZAP70-saFRET biosensors with active kinase domain (KA) or dead kinase domain (KD) before and after TAK-659 treatment. Scale bars, 10 µm. The color bar indicates ECFP/FRET emission ratio, with hot and cold colors representing the high and low ratios, respectively.

Title: Supplementary Video 4:

Description: Time course of the parental and improved ZAP70 biosensors before and after TAK-659 treatment. Scale bars, 10 µm. The color bar indicates ECFP/FRET ratio, with hot and cold colors representing the high and low ratios, respectively.

Title: Supplementary Video 5:

Description: Time course of improved or WT biosensors before and after TCR activation induced by CD3/CD28 antibody stimulation in Jurkat or P116 cells. Scale bars, 10 µm.

Title: Supplementary Video 6:

Description: Time course of CAR-T cell expressing the optimized ZAP70 biosensor before and after the engagement with a tumor cell. Scale bars, 10 µm.

Title: Supplementary Video 7:

Description: Time course of FRET ratio change in HEK293 cells with improved ZAP70 saFRET biosensor before and after different inhibitor treatment. The TAK-659 (10µM) was used as the negative control, which cannot sufficiently inhibit the ZAP70 kinase. Scale bars, 10 µm.
